# Research progress on nutritional support for patients with liver cirrhosis complicated by upper gastrointestinal bleeding

**DOI:** 10.3389/fnut.2025.1727092

**Published:** 2025-12-11

**Authors:** Yun Dong, Pei-Yue Chen, Chuan You, Jing-Dong Li, Song-Tao Chen

**Affiliations:** 1 Department of Hepatobiliary Surgery, Affiliated Hospital of North Sichuan Medical College, Nanchong, China; 2 Sichuan Branch of National Clinical Research Center for Digestive Diseases, Affiliated Hospital of North Sichuan Medical College, Sichuan Clinical Research Center for Digestive Diseases‌, Nanchong, China; 3 Party Committee Office, North Sichuan Medical College Affiliated Hospital, Nanchong, China

**Keywords:** liver cirrhosis, upper gastrointestinal bleeding(UGIB), nutritional support, nutritional risk screening, parenteral nutrition, enteral nutrition, research progress

## Abstract

Upper gastrointestinal bleeding (UGIB) in patients with liver cirrhosis represents a common clinical emergency characterized by sudden onset and high mortality. The prognosis of these patients is strongly correlated with their nutritional status. Malnutrition serves as a key determinant of clinical outcomes, making nutritional support a critical component of comprehensive care. Early identification and timely intervention can effectively prevent disease progression, reduce complication rates, and improve both treatment efficacy and survival. This article synthesizes evidence from previous research to summarize nutritional support strategies for cirrhotic patients with upper gastrointestinal bleeding(UGIB). It addresses nutritional screening tools, routes of administration, and fundamental principles of nutritional support, with the aim of offering a reference for clinical management.

## 1 Introduction

Liver cirrhosis represents the end-stage pathological manifestation of chronic liver disease, with common etiologies including viral hepatitis, autoimmune disorders, cholestatic diseases, obesity, and chronic alcohol abuse. In recent years, both the incidence and mortality of cirrhosis have been rising globally. Epidemiological data indicate that over 1 million people worldwide die from cirrhosis each year (1) If not treated in a timely manner, cirrhosis can lead to severe complications such as UGIB, hepatic encephalopathy, primary liver cancer, ascites, and infections. Among these, upper gastrointestinal bleeding is one of the most critical emergencies in cirrhotic patients. It is defined as bleeding originating from the upper digestive tract—including the esophagus, stomach, and duodenum—and poses serious health risks, potentially becoming life-threatening. Patients with cirrhosis are often in a hypermetabolic state and experience impaired nutrient absorption, resulting in inadequate intake and increased energy consumption, which further exacerbates nutritional stress. During an acute bleeding episode, symptoms such as hematemesis and melena may occur; in severe cases, hemorrhagic shock can ensue (2, 3). Studies report that the prevalence of malnutrition in patients with cirrhosis and UGIB exceeds 60% (4). Nutritional support therapy is therefore a vital component of management, helping to maintain nutritional status and improve prognosis. Recent advances in nutritional support have been made in the areas of assessment tools and interventional strategies, including parenteral and enteral nutrition. This article aims to review nutritional support strategies for patients with cirrhosis complicated by UGIB, providing clinicians with a reference to better understand and address nutritional challenges in this population.

### 1.1 Liver dysfunction and synthetic disorders

Liver dysfunction and impaired synthesis capacity are hallmark features of decompensated cirrhosis. As liver function deteriorates, the synthesis of essential plasma proteins such as albumin and coagulation factors is significantly reduced. The decline in hepatocyte count and functional impairment further disrupt key physiological processes, including protein synthesis, glycogen storage, and lipid metabolism. A prospective observational study by Crisan et al. (5). demonstrated that patients with decompensated cirrhosis exhibit a high prevalence of malnutrition and poor survival outcomes, with an overall mortality rate as high as 70%. Notably, patients classified as Child-Pugh class C, characterized by severely compromised hepatic reserve, often present with marked hypoalbuminemia and coagulation disorders, which further exacerbate the progression of malnutrition.

### 1.2 Portal hypertension and gastrointestinal dysfunction

Portal hypertension serves as a key pathophysiological basis for gastrointestinal dysfunction in cirrhotic patients. Sustained elevation of portal pressure induces mucosal congestion and edema of the gastrointestinal tract, and may even lead to submucosal arteriovenous shunting, severely compromising the digestion and absorption of nutrients. Intestinal mucosal edema directly reduces the available absorptive surface area and markedly diminishes brush border enzyme activity, thereby impairing the efficiency of carbohydrate, protein, and lipid assimilation. Furthermore, portal hypertension promotes gut dysbiosis and bacterial overgrowth, which exacerbates intestinal barrier injury, accelerates endotoxin translocation, and triggers systemic inflammatory responses (6, 7).

### 1.3 The vicious cycle of bleeding and malnutrition

Pre-existing malnutrition in patients with UGIB further compromises the protective and self-repair capacity of the gastrointestinal mucosa. Inadequate protein-energy supply directly impairs the availability of essential substrates—such as zinc and vitamin A—required for mucosal cell regeneration and repair. Concurrent deficiencies in micronutrients undermine mucosal barrier integrity, which can exacerbate bleeding, prolong its duration, and ultimately perpetuate a vicious cycle of bleeding, malnutrition, and rebleeding (8, 9). Studies indicate that patients with both UGIB and malnutrition face a significantly elevated risk of rebleeding and bleeding-related mortality (10).

### 1.4 Digestive and absorptive dysfunction

Beyond the direct effects of hemorrhage, patients with liver cirrhosis frequently exhibit a range of digestive impairments. Following a bleeding episode, gastric acid and bile secretion are often suppressed, further compromising the digestion and absorption of nutrients in the stomach and small intestine (11). Additionally, abnormal intestinal peristalsis and pancreatic exocrine insufficiency further contribute to malabsorption. The interplay of these factors results in inadequate nutrient absorption and utilization, even with sufficient oral intake, ultimately impairing normal physiological function. This malabsorptive state affects not only macronutrients but also critical micronutrientes including iron, calcium, zinc, and fat-soublevitamins—predisposing patients to multiple nutritional deficiencies (10). Therefore, in individuals with cirrhosis, particularly those complicated by upper gastrointestinal bleeding, the implementation of a scientifically balanced diet and individualized nutritional support constitutes a key therapeutic strategy to interrupt this vicious cycle and improve clinical outcomes.

## 2 Nutritional screening and assessment tools

Timely nutritional screening and assessment are critical for identifying and intervening in malnutrition among patients with cirrhosis complicated by upper gastrointestinal bleeding. A comprehensive evaluation system should integrate objective anthropometric and laboratory measures with standardized screening tools. Objective indicators—such as BMI, mid-upper arm circumference, triceps skinfold thickness, serum albumin, and prealbumin—provide a quantitative basis for nutritional evaluation. In addition, validated nutritional risk assessment tools recommended by the European Society for Clinical Nutrition and Metabolism should be applied. These include the Nutritional Risk Screening(NRS 2002), Royal Free Hospital Nutritional Prioritizing Tool (RFH-NPT), Malnutrition Universal Screening Tool (MUST), Patient-Generated Subjective Global Assessment (PG-SGA), Liver Disease Undernutrition Screening Tool (LDUST), and Prognostic Nutritional Index(PNI), among others (12–14). By synthesizing information on nutritional status, disease severity, and dietary intake, these instruments facilitate the identification of high-risk individuals and support the development of tailored nutritional intervention strategies.

### 2.1 Anthropometric indicators

#### 2.1.1 BMI

Body mass index (BMI) is widely used as a screening tool for protein-energy malnutrition. It is calculated as weight in kilograms divided by the square of height in meters. According to Chinese criteria, a BMI ≤ 18.5 indicates underweight, ≥24 indicates overweight, and ≥28 indicates obesity. However, the accuracy of BMI is substantially limited in cirrhotic patients with ascites, pleural effusion, or peripheral edema. Fluid retention in these individuals leads to artificially elevated body weight, rendering BMI an unreliable indicator of true tissue mass. As a result, its use may mask underlying malnutrition and yield false-negative results in nutritional screening (15, 16). Therefore, nutritional risk assessment in this population should incorporate alternative indicators unaffected by fluid and sodium retention to ensure a comprehensive evaluation.

#### 2.1.2 AC and TSF

Mid-upper arm circumference (AC) and triceps skinfold thickness (TSF) serve as key indicators for evaluating body composition. AC is measured at the midpoint between the acromion and the olecranon, reflecting combined muscle and adipose tissue content, whereas TSF specifically estimates total body fat mass. According to data from Modern Clinical Nutrition on adults in northern China, the mean AC is approximately 27.3 cm for men aged 26–45 years and 26.3 cm for women (17). In patients with liver cirrhosis, AC and TSF hold particular clinical value. These measurements help mitigate assessment inaccuracies resulting from fluid overload—such as ascites or edema—that often distort body weight readings. Consequently, they more reliably reflect true muscle and fat reserves. Given their simplicity, cost-effectiveness, and non-invasive nature, AC and TSF are recommended for inclusion in the routine nutritional assessment of cirrhotic patients (18, 19).

### 2.2 Laboratory indicators

Albumin is a traditional indicator for assessing long-term nutritional status and liver reserve function. Changes in its concentration are closely related to the prognosis of patients with liver cirrhosis (20). Its half-life is about 20 days, and a decrease in its level often indicates a continuous decline in hepatic synthetic function, which is an important sign that liver function has entered decompensation. Clinical observations have found that hypoalbuminemia is positively correlated with the risk of complications such as ascites and infections, and it is also an independent risk factor for predicting patient survival. Prealbumin has a half-life of about 2–3 days, and its sensitivity to short-term changes theoretically makes it suitable for monitoring the effects of nutritional support, but it also makes its levels susceptible to interference from various factors (21). In the acute phase of cirrhosis complicated by upper gastrointestinal bleeding, the prealbumin level may reflect the degree of stress and inflammatory response more than merely representing nutritional status (22). Electrolyte disturbances, changes in dietary status, and the liver’s own functional reserve can all affect the synthesis and metabolism of prealbumin. In actual clinical practice, it is recommended to analyze these two indicators in conjunction with other clinical data. Albumin levels are more suitable as a basis for long-term prognosis, while dynamic changes in prealbumin may help assess the effects of short-term interventions, although confounding factors such as inflammation need to be excluded. This comprehensive assessment approach helps to more accurately understand the patient’s true condition and provides more valuable reference for treatment decision-making.

### 2.3 The Child-Pugh classification of liver cirrhosis

The Child-Pugh classification assesses liver function using five clinical indicators: serum bilirubin, albumin, prothrombin time prolongation, ascites, and hepatic encephalopathy. Based on a composite score, patients are categorized into Class A (5–6 points), Class B (7–9 points), or Class C (10–15 points) (23). This grading system is valuable for predicting short-term prognosis in patients with cirrhosis, as higher scores indicate more severe liver dysfunction and poorer clinical outcomes. However, the Child-Pugh classification has limitations, particularly in its reliance on subjective clinical assessments for ascites and hepatic encephalopathy, which lack standardized objective criteria. Therefore, in clinical practice, it is recommended to complement this tool with additional approaches, such as the Model for End-Stage Liver Disease (MELD) score, imaging studies, and dynamic monitoring of laboratory parameters (24).

### 2.4 NRS2002

The Nutritional Risk Screening 2002 (NRS2002), recommended by the European Society for Clinical Nutrition and Metabolism (ESPEN) and the American Society for Parenteral and Enteral Nutrition (ASPEN) (25, 26), is a widely used tool for screening nutritional risk in hospitalized patients aged 18 to 90. It comprises three components: disease severity, impaired nutritional status, and age. The total score from these dimensions determines nutritional risk. A score of ≥3 indicates malnutrition or nutritional risk, necessitating nutritional support, whereas a score <3 requires only weekly reassessment (27). This tool is simple, non-invasive, and highly operable. However, it has certain limitations; for example, weight measurement—a key element—is often inaccurate in bedridden patients or those with ascites and edema. Recent clinical studies have validated its applicability in patients with cirrhosis and upper gastrointestinal bleeding. Lou et al. (28) applied NRS2002 in 115 cirrhotic patients with portal hypertension who underwent endoscopic tissue adhesive injection for esophagogastric variceal bleeding and found that the risk of post-endoscopic infection was associated with nutritional risk level. In a comparative study by Zhang et al. (29), NRS2002 demonstrated a sensitivity of 66.67% and a specificity of 93.33% in nutritional screening for cirrhotic patients.

### 2.5 RFH-NPT

The Royal Free Hospital Nutritional Prioritizing Tool (RFH-NPT) is a nutritional screening instrument specifically developed for cirrhotic patients, recommended by several UK research centers. It offers distinct advantages in clinical practice, including straightforward administration and rapid assessment. The tool classifies patients into three risk categories within 3 min through a three-step process: determining the presence of severe acute alcohol-related hepatitis or ongoing tube feeding, assessing fluid retention status, and calculating a total score. Risk stratification is defined as low (0 points), medium (1 point), or high (2–7 points) (30). Developed in accordance with current nutritional science and clinical practice guidelines, RFH-NPT incorporates the distinct pathophysiology of cirrhosis and practical clinical experience, ensuring reliable and applicable assessment results (31–34). A 2025 network meta-analysis of 10 studies (*n* = 1,299) demonstrated that the Royal Free Hospital Nutrition Prioritizing Tool (RFH-NPT) achieved the highest sensitivity of 93% for nutritional risk identification, making it highly suitable for initial screening to minimize missed diagnoses. Despite its moderate specificity of 72%, the RFH-NPT remains the preferred tool for nutritional screening in patients with liver cirrhosis, given its superior performance in correctly identifying at-risk individuals (35).

### 2.6 PG-SGA

The Patient-Generated Subjective Global Assessment (PG-SGA) is a comprehensive nutritional assessment tool that integrates patient-reported subjective information with objective clinical evaluation. It encompasses five key components: changes in body weight, dietary intake, gastrointestinal symptoms, functional capacity, and physical examination findings. Based on the synthesized medical history and physical assessment, patients are categorized into one of three nutritional grades: A (well-nourished), B (moderately or suspected malnourished), or C (severely malnourished) (36, 37). Studies have demonstrated that the PG-SGA exhibits a sensitivity of 77.2% and a specificity of 88.2% (38). It shows good applicability in identifying malnutrition among patients with liver cirrhosis. A key limitation of this tool is its dependence on subjective symptoms and routine physical examination, which lacks objective measures of muscle mass. Consequently, it may underestimate the prevalence of malnutrition, particularly in patients with sarcopenia (39).

### 2.7 MUST

The Malnutrition Universal Screening Tool (MUST), developed by the British Association for Parenteral and Enteral Nutrition (BAPEN), is widely used in clinical and community healthcare settings to rapidly identify individuals at risk of malnutrition. It evaluates three parameters—body mass index (BMI), recent unintentional weight loss, and the impact of acute disease—to categorize patients into low (0 points), medium (1 point), or high (≥2 points) malnutrition risk (40). Although MUST has limitations in patients with cirrhosis, as ascites can distort weight and BMI measurements, it remains applicable for initial screening. Studies indicate that MUST has a high specificity of 91.4%, accurately ruling out non-malnourished patients; however, its low sensitivity (43.0%) suggests a substantial risk of underdiagnosis, failing to identify nearly half of those truly malnourished (35).

### 2.8 LDUST

The Liver Disease Undernutrition Screening Tool (LDUST) was jointly developed by the American Society for Parenteral and Enteral Nutrition (ASPEN) and the Academy of Nutrition and Dietetics (AND) as a nutrition screening instrument specifically for patients with chronic liver disease lasting 3 months or longer. It is designed to identify malnutrition risk and inform subsequent clinical interventions (41). LDUST is simple to administer and provides a rapid assessment, covering six primary domains: weight changes, dietary intake, muscle wasting, fluid retention, and functional status decline (42). A cross-sectional observational study reported that the Liver Disease Undernutrition Screening Tool (LDUST) demonstrated high sensitivity (92.1%) but moderate specificity (67.2%) (31). However, as LDUST relies entirely on patient self-reporting without incorporating objective measures, it is susceptible to subjective bias. Therefore, while it is useful for initial screening, its results should be interpreted in conjunction with physical examination and laboratory data to improve diagnostic accuracy.

### 2.9 PNI

The Prognostic Nutritional Index (PNI) is a composite metric integrating nutritional and immunological status, calculated as follows: PNI = 5 × platelet count (10^9^/L) + serum albumin (g/L) (43). Evidence supports its utility in evaluating disease severity and nutritional status in patients with liver cirrhosis (44, 45). In a study of 513 cirrhotic patients, PNI was identified as an independent prognostic factor for decompensated cirrhosis, demonstrating excellent predictive performance for mortality risk, with an area under the curve (AUC) of 0.897 (44). Clinically, lower PNI values are associated with increased risks of complications—including upper gastrointestinal bleeding, hepatic encephalopathy, and infections—and poorer overall prognosis. Originally developed to assess nutritional status and surgical risk, PNI has since been widely adopted in various medical fields such as oncology, respiratory medicine, and cardiology, establishing itself as a significant prognostic biomarker (Table 1) (46).

**Table 1:** Comparison of nutritional assessment tools.

Assessment tool	Applicable people	Advantages	Limitations	Sensitivity/Specificity
BMI	General population.	Simple, widely applicable; useful for initial screening of protein-energy malnutrition.	Accuracy is compromised by fluid retention (ascites, edema), leading to false negatives.	No well-validated sensitivity/specificity data available.
AC + TSF	Patients with cirrhotic ascites.	Non-invasive, low-cost; unaffected by fluid overload; provides a reasonable estimate of muscle and fat mass.	Operator-dependent, with potential inter-observer variability;	No well-validated sensitivity/specificity data available.
Albumin	Cirrhotic patients evaluated for long-term nutritional status and liver reserve function.	A traditional biomarker for assessing long-term nutritional status and liver synthetic function; serum levels correlate with prognosis in cirrhosis.	Has a long half-life and cannot quickly reflect acute nutritional status.	No well-validated sensitivity/specificity data available.
Prealbumin	Patients requiring short-term monitoring of nutritional support outcomes.	Short half-life allows for monitoring short-term nutritional changes and response to intervention.	Levels are confounded by inflammation, stress, and liver dysfunction; not solely reflective of nutritional status.	No well-validated sensitivity/specificity data available.
Child-Pugh classification	Patients with liver cirrhosis.	Predictable short-term prognosis.	The assessment of ascites and hepatic encephalopathy relies on subjective clinical judgment and lacks standardized objective criteria.	No well-validated sensitivity/specificity data available.
NRS2002	Hospitalized patients aged 18–90.	Comprehensive, assessing disease severity, nutritional status, and age; widely recommended and standardized.	May be insufficient for identifying nutritional risk in patients with advanced liver cirrhosis.	Sensitivity: 66.67% Specificity: 93.33%
RFH-NPT	Patients with liver cirrhosis.	User-friendly; an independent predictor of survival and liver function deterioration in cirrhosis.	Applicability in non-alcoholic liver disease is less clear; relies on clinical judgment, introducing subjectivity.	Sensitivity: 93% Specificity: 72%
PG-SGA	Patients with chronic diseases.	Integrates subjective and objective measures; well-suited for patients with chronic diseases like cirrhosis.	Relying on the patient’s subjective report.	Sensitivity: 77.2% Specificity: 88.2%
MUST	Community, outpatient, and inpatient patients.	Quick and simple.	Not suitable for patients with fluid retention.	Sensitivity: 43% Specificity: 91.4%
LDUST	Patients with chronic liver disease for ≥3 months.	Specifically designed for chronic liver disease; simple and rapid to administer.	Based entirely on subjective patient report, lacks objective measures, and is susceptible to bias.	Sensitivity: 92.1% Specificity: 67.2%
PNI	Patients with multiple diseases.	Provides a quantitative score combining nutritional and immune status.	Relies on laboratory data and is influenced by inflammatory states.	No well-validated sensitivity/specificity data available.

## 3 Principles of nutritional support

In 2019, the European Association for the Study of the Liver (EASL)published clinical practice guidelines on nutritional management in chronic liver disease, highlighting that patients with a BMI < 18.5 kg/m^2^, Child-Pugh class C status, or decompensated cirrhosis are at elevated risk of malnutrition (15). The guidelines emphasize the importance of nutritional support for cirrhotic patients experiencing upper gastrointestinal bleeding. During the acute bleeding phase, the patient must strictly fast and maintain bodily needs through intravenous nutrition. Once hemodynamic stability is achieved and bleeding is controlled, a transition to enteral nutrition should be initiated as early as clinically feasible. Evidence indicates that energy requirements are generally elevated in cirrhosis, with total daily energy expenditure estimated at 1.3–1.4 times resting energy expenditure (47). Nutritional recommendations include daily intakes of 30–35 kcal/kg and 1.2–1.5 g/kg of protein for malnourished cirrhotic patients (48), while critically ill patients should receive no less than 35–40 kcal/kg per day (15, 22, 49). Dietary advancement should follow a stepwise and safety-focused approach, progressing from liquid to semi-solid consistencies. Foods should be soft, easily digestible, and non-irritating to avoid mucosal injury and rebleeding. Small, frequent meals are recommended throughout. The entire nutritional support process requires professional supervision and close monitoring to prevent complications such as hepatic encephalopathy, support recovery, and enhance quality of life (50, 51). In addition, nocturnal nutritional supplementation in patients with cirrhosis can help improve body mass index, increase lean body mass, and reduce the risks of ascites and hepatic encephalopathy (18).

### 3.1 Parenteral nutrition (PN)

Parenteral nutrition (PN) involves the intravenous administration of nutrients via central or peripheral venous access to treat or prevent severe malnutrition in patients unable to tolerate or receive adequate oral or enteral nutrition (52). According to the 2020 ESPEN practical guidelines, PN is indicated for cirrhotic patients when oral or enteral nutrition is ineffective or unfeasible (48), and should be initiated when enteral nutrition fails to meet nutritional needs (30). Upper gastrointestinal bleeding often compromises nutritional intake, making tube feeding or intravenous nutrition necessary in clinical practice. In cirrhosis, upper gastrointestinal bleeding arises from multiple etiologies, primarily esophageal variceal rupture, portal hypertensive gastropathy (PHG), hepatogenic peptic ulcer, acute gastric mucosal lesions, gastritis, and reflux esophagitis. Esophageal variceal hemorrhage represents the most frequent cause. During active bleeding, oral intake may stimulate gastrointestinal secretions, enhance motility, and increase portal venous blood flow, thereby elevating the risk of continued bleeding. Consequently, it is recommended that patients refrain from eating and drinking and be hospitalized for at least 48–72 h after endoscopic treatment. (53, 54). PN during this period provides essential energy and nutrients while reducing hepatic and gastrointestinal metabolic, thereby minimizing rebleeding risk (26, 55). In a randomized controlled trial by Xianfen et al. (56) 40 patients with cirrhotic portal hypertension received postoperative PN supplemented with arginine, demonstrating significant improvements in both nutritional and immune parameters. Similarly, a retrospective study of 208 cirrhotic patients with acute variceal bleeding managed exclusively with PN compared 86 recipients of *ω*-3 fatty acid-enriched PN with 122 controls receiving standard PN. The ω-3 enriched group had a significantly shorter mean hospital stay (7.9 ± 4.2 days) versus controls (10.7 ± 7.3 days) (57).

### 3.2 Enteral nutrition (EN)

**Figure 1:**
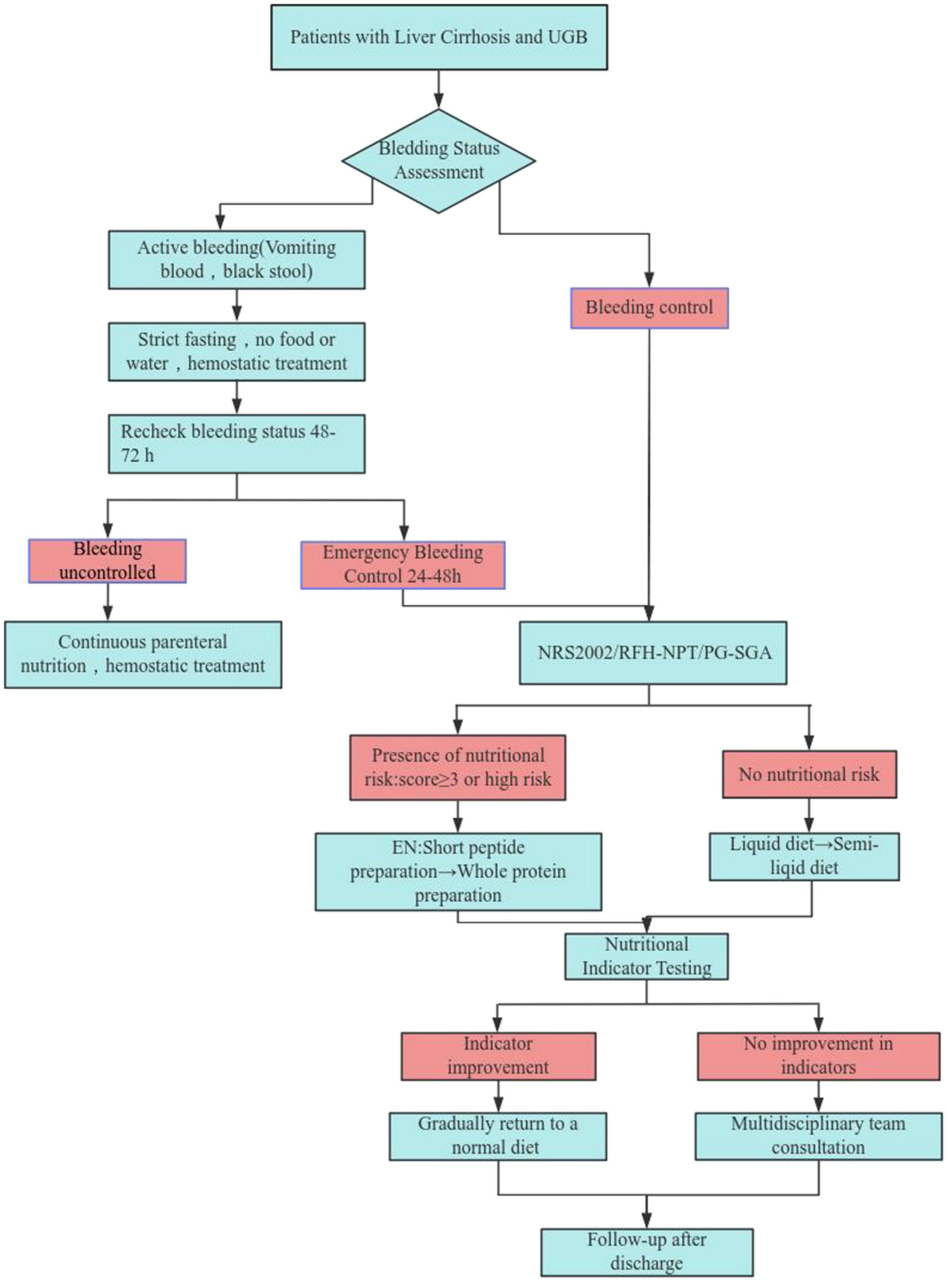
Nutrition management decision flowchart.

Enteral nutrition refers to the delivery of nutritional support via oral intake or feeding tubes to maintain or enhance a patient’s nutritional status (58). This modality administers comprehensive nutrients—including carbohydrates, amino acids, lipids, vitamins, and minerals—and is characterized by its cost-effectiveness and ease of administration. EN is typically indicated when patients cannot adequately meet energy and metabolic requirements through regular diet alone (59). Formula selection should be individualized according to gastrointestinal function. For patients in unstable condition or during early recovery, short-peptide formulations such as Peptamen, Peptison, are recommended. As gastrointestinal function recovers and stabilizes, transition to whole-protein formulas such as Ensure, Nutrison, Nutrison powder are appropriate. The timing of EN initiation should be guided by clinical status rather than a fixed protocol. In cirrhotic patients, enteral support may be gradually introduced 24–48 h after the last bleeding episode, with stool color serving as a practical reference (30). The meta-analysis by Obeidat et al. (60), which included 10 randomized controlled trials (RCTs) with 1,051 patients, demonstrated that early EN significantly reduces the length of hospital stay compared to delayed EN. Figure 1 Nutrition management decision flowchart.

### 3.3 Oral nutritional supplements(ONS)

Oral nutritional supplements (ONS) are a form of nutritional support in which specially formulated medical foods are consumed orally in addition to the regular diet to provide supplemental energy, protein, fat, vitamins, and minerals (61). For patients with liver cirrhosis and UGIB who are clinically stable and have resumed oral intake—yet cannot meet their elevated metabolic needs through diet alone—ONS offer a convenient, non-invasive, and physiologically appropriate first-line nutritional intervention. In a prospective study by Wang et al. (62) involving 197 patients, the experimental group received oral nutrition 4 h after endoscopic treatment, whereas the control group remained fasting for over 48 h with parenteral nutrition support. Results demonstrated that early oral nutrition did not increase the risk of rebleeding or mortality. Similarly, a study by Sidhu et al. (63)confirmed that early oral feeding in this population effectively reduces the incidence of early onset infections. Therefore, once bleeding is controlled, patients should be transitioned gradually from enteral nutrition to oral nutritional supplementation.

## 4 New strategies for nutritional support

### 4.1 Nutritional regulation based on the microbiome

Gut microbiota dysbiosis plays a significant role in the pathogenesis of malnutrition and rebleeding in patients with liver cirrhosis. In recent years, probiotics—primarily containing Bifidobacterium and Lactobacillus strains—have become a focus of microecological intervention research (64). Their mechanisms of action include replenishing beneficial intestinal bacteria and optimizing microbial composition, thereby enhancing intestinal barrier function, reducing endotoxin translocation into the bloodstream, and modulating immune and inflammatory responses (65). A meta-analysis by Xia et al., which included 33 randomized controlled trials, further confirmed that probiotic supplementation significantly improves liver function-related inflammatory markers and other biochemical parameters in patients with cirrhosis (66). However, there remains a lack of large-scale, high-quality clinical studies in this field. Future research should aim to clarify the optimal timing, strain selection, formulation, and dosage regimens for probiotic interventions to facilitate standardized and personalized clinical applications.

### 4.2 Multidisciplinary collaboration

A multidisciplinary team (MDT) adopts a patient-centered, collaborative approach that integrates expertise from various disciplines to provide comprehensive and individualized diagnosis and treatment. This model is well established in the management of complex conditions such as oncology, major trauma, and cardiovascular diseases (67–69). Accordingly, nutritional management in patients with cirrhosis complicated by upper gastrointestinal bleeding should involve close collaboration among hepatology, nutrition, and nursing teams (70). Within this framework, the hepatology team is responsible for disease assessment and foundational treatment, including evaluation of liver function and bleeding risk. The nutrition team formulates individualized nutrition plans based on screening results and dynamically adjusts energy and nutrient provision. The nursing team ensures implementation, conducts ongoing monitoring, and provides patient education to facilitate effective execution of the care plan.

## 5 Conclusion

Nutritional support therapy holds significant clinical importance in the management of patients with cirrhosis complicated by UGIB. The early identification of malnutrition and the implementation of appropriate nutritional interventions can improve nutritional status, reduce the incidence of complications, and enhance both survival rates and quality of life. In clinical practice, standardized nutritional screening tools should be utilized for systematic evaluation, taking into account liver function grade, BMI, and other relevant parameters to formulate individualized nutritional support plans. The mode of nutritional intervention should be selected based on the patient’s clinical phase. During acute bleeding stages, PN is recommended; once the patient is stabilized, a transition to EN or ONS should be initiated as early as possible to ensure adequate nutrient intake. Future research should aim to develop more precise nutritional assessment tools and targeted interventions. A multidisciplinary team involving clinicians, nutritionists, and nurses should collaborate to establish more rational nutritional support strategies, thereby providing a stronger scientific foundation for the comprehensive management of cirrhosis. Long-term nutritional monitoring and regular reassessment of discharged patients are essential to screen for malnutrition risk and tailor interventions in a timely manner, thereby optimizing long-term outcomes.
